# Meta-analysis of human cancer microarrays reveals GATA3 is integral to the estrogen receptor alpha pathway

**DOI:** 10.1186/1476-4598-7-49

**Published:** 2008-06-04

**Authors:** Brian J Wilson, Vincent Giguère

**Affiliations:** 1 Molecular Oncology Group, Room H5-45, McGill University Health Centre, 687 Pine Avenue West, Montréal, Québec, H3A 1A1, Canada

## Abstract

**Background:**

The transcription factor GATA3 has recently been shown to be necessary for mammary gland morphogenesis and luminal cell differentiation. There is also an increasing body of data linking GATA3 to the estrogen receptor α (ERα) pathway. Among these it was shown that GATA3 associates with the promoter of the ERα gene and ERα can reciprocally associate with the GATA3 gene. GATA3 has also been directly implicated in a differentiated phenotype in mouse models of mammary tumourigenesis. The purpose of our study was to compare coexpressed genes, by meta-analysis, of GATA3 and relate these to a similar analysis for ERα to determine the depth of overlap.

**Results:**

We have used a newly described method of meta-analysis of multiple cancer studies within the Oncomine database, focusing here predominantly upon breast cancer studies. We demonstrate that ERα and GATA3 reciprocally have the highest overlap with one another. Furthermore, we show that when both coexpression meta-analysis lists for ERα and GATA3 are compared there is a significant overlap between both and, like ERα, GATA3 coexpresses with ERα pathway partners such as pS2 (*TFF1*), *TFF3*, *FOXA1*, *BCL2*, *ERBB4*, *XBP1*, *NRIP1*, *IL6ST*, keratin 18(*KRT18*) and cyclin D1 (*CCND1*). Moreover, as these data are derived from human tumour samples this adds credence to previous cell-culture or murine based studies.

**Conclusion:**

GATA3 is hypothesized to be integral to the ERα pathway given the following: (1) The large overlap of coexpressed genes as seen by meta-analysis, between GATA3 and ERα, (2) The highest coexpressing gene for GATA3 was ERα and *vice-versa*, (3) GATA3, like ERα, coexpresses with many well-known ERα pathway partners such as pS2.

## Background

While GATA3 has most intensively been studied in the immune system [1] GATA3 is also expressed in other biological environments such as the human endometrium epithelial cells, where levels are regulated in a cyclic manner [2]. GATA3 levels are also considered a good prognostic biomarker in breast tumours. Specifically, in the luminal A subtype of breast cancer GATA3 has both a favorable prognostic outcome, and the highest ERα and GATA3 expression of all breast tumours [3]. Consistent with this, basal-like tumours have the lowest GATA3 expression and the worst prognosis. GATA3 has also been shown in murine models to be essential to the development and maintenance of mammary luminal cells [4,5]. There is also tentative data showing that different polymorphisms of the GATA3 gene may associate with differential susceptibility to breast cancer [6].

GATA3 levels have previously been correlated with expression of ERα [7] and both were shown to reciprocally regulate one another at the transcriptional level in a cell-culture based system in a cross-regulatory loop [8]. Furthermore, in a meta-analysis of ERα 10 genes were proposed as classifier of ERα positive breast tumours, listing GATA3 as one of these [9]. A study has also compared microarray experiments between estradiol-induced genes from MCF-7 cells, and transfected GATA3-induced genes from 293T cells to assess common upregulated genes [10].

In an elegant series of experiments utilizing MMTV-PyMT (polyoma middle T antigen) mice it was first shown that GATA3 expression was downregulated with the transition from adenoma to carcinoma in mammary tumours, and the expression was lost in lung metastases. Infection of the MMTV-PyMT carcinomas with GATA3 upregulated markers of differentiation and resulted in a dramatic 27-fold reduction in lung metasases [11]. Further crossing of these mice with an inducible cre-WAP (whey acidic protein – specific to luminal mammary epithelial cells) driven knockout of *GATA3*, resulted in loss of markers of terminal differentiation, detachment from the basal membrane and apoptosis. This is consistent with the requirement of GATA3 in differentiated tumours.

As described in a recent study known pathway partners have been shown to yield a similar 'meta-analysis coexpression signature' i.e. having a significant overlap of coexpressed genes can link proteins to the same pathways [12]. Thus performing independent meta-analyses for ERα and GATA3 (putative pathway partners), then comparing the results for overlapping genes would yield a highly significant number of genes if these transcription factors were in the same pathway. We report here not only that these meta-analyses have a high degree of overlap, but that genes identified are consistent with previous reports of the ERα pathway regulation. Additionally we show this correlation with previously identified ERα target genes by combining our meta-analysis data with both RT-PCR and genome-wide location analysis from other studies. These data not only confirm GATA3 as being a key player in the ERα pathway, but also give fresh insights into the pathway itself.

## Methods

### Meta-analysis

The following procedure was undertaken for independent meta-analyses of GATA3 or ERα: a co-expression gene search was performed within Oncomine [13]. Twenty-one studies were chosen for analysis, most of which were breast cancer studies. The top 400 coexpressed genes were extracted and filtered to give one representative gene per study (removing duplicates and ESTs). These filtered genelists were then compared for repeating coexpressed genes over multiple studies. The frequency cutoff was 3 studies (14% of 21 studies). This generated a meta-analysis list for ERα or GATA3, which were then compared for overlap. As the overlap was high the stringency was increased to 4 studies (19%), the data of which is used for Table 1. Gene names were obtained using Genecards [14].

**Table 1:** Overlapping meta-analyses of GATA3 and ERα at cutoff of 4 studies (19%)

**Overlap of ERα and GATA3 (4 or more studies)**			
ERα = 257, GATA3 = 194, OVERLAP = 108			
	**ERα**	**GATA3**	
GATA3	**48%**	100%	GATA binding protein 3
ESR1	100%	**67%**	estrogen receptor 1 (estrogen receptor alpha)
XBP1	**38%**	**52%**	X-box binding protein 1
FOXA1	**33%**	**52%**	forkhead box A1
FOXC1	19%	24%	forkhead box C1
TFF1	**33%**	**52%**	trefoil factor 1 (breast cancer, estrogen-inducible sequence expressed in) [pS2]
TFF3	**38%**	**67%**	trefoil factor 3 (intestinal)
NRIP1	19%	19%	nuclear receptor interacting protein 1 (RIP140)
BCL2	**43%**	**67%**	B-cell CLL/lymphoma 2
ACADSB	**38%**	**48%**	acyl-Coenzyme A dehydrogenase, short/branched chain
LAF4	**43%**	**38%**	lymphoid nuclear protein related to AF4
COX6C	**38%**	**33%**	cytochrome c oxidase subunit VIc
FBP1	**38%**	**33%**	fructose-1,6-bisphosphatase 1
IGF1R	**38%**	**33%**	insulin-like growth factor 1 receptor
IRS1	**33%**	**33%**	insulin receptor substrate 1
CELSR2	**38%**	**38%**	cadherin, EGF LAG seven-pass G-type receptor 2 (flamingo homolog, Drosophila)
LRBA	**38%**	**38%**	LPS-responsive vesicle trafficking, beach and anchor containing
NAT1	**33%**	**57%**	N-acetyltransferase 1 (arylamine N-acetyltransferase)
SCNN1A	**38%**	**57%**	sodium channel, nonvoltage-gated 1 alpha
DNAJC12	**33%**	**48%**	DnaJ (Hsp40) homolog, subfamily C, member 12
RAB31	**38%**	19%	RAB31, member RAS oncogene family
RABEP1	**33%**	**43%**	rabaptin, RAB GTPase binding effector protein 1
SELENBP1	**33%**	**33%**	selenium binding protein 1
FAAH	**38%**	**33%**	fatty acid amide hydrolase
TNFSF10	**38%**	**33%**	tumor necrosis factor (ligand) superfamily, member 10
SLC22A18	**33%**	24%	solute carrier family 22 (organic cation transporter), member 1
SLC39A6	**38%**	**57%**	solute carrier family 39 (zinc transporter), member 6 (Estrogen regulated protein LIV-1)
SLC40A1	**33%**	19%	solute carrier family 40 (iron-regulated transporter), member 1
SLC9A3R1	19%	**43%**	solute carrier family 9 (sodium/hydrogen exchanger), isoform 3 regulator 1
SIAH2	**33%**	**33%**	seven in absentia homolog 2 (Drosophila)
SERPINA3	**38%**	24%	serpin peptidase inhibitor, clade A (alpha-1 antiproteinase, antitrypsin), member 3
SERPINA5	**33%**	19%	serine (or cysteine) proteinase inhibitor, clade A (alpha-1 antiproteinase, antitrypsin), 5
SERPINA6	19%	24%	serine (or cysteine) proteinase inhibitor, clade A (alpha-1 antiproteinase, antitrypsin), 6
ERBB3	**33%**	19%	v-erb-b2 erythroblastic leukemia viral oncogene homolog 3 (avian)
ERBB4	19%	**48%**	v-erb-a erythroblastic leukemia viral oncogene homolog 4 (avian)
IL6ST	24%	**38%**	interleukin 6 signal transducer (gp130, oncostatin M receptor)
KIAA0040	24%	24%	KIAA0040 protein
KIAA0303	**33%**	**43%**	Similar to Mouse serine/threonine protein kinase MAST205
KIAA0882	19%	19%	KIAA0882 protein
ITPR1	24%	**33%**	inositol 1,4,5-triphosphate receptor, type 1
INPP4B	24%	**43%**	inositol polyphosphate-4-phosphatase, type II, 105kDa
JMJD2B	24%	**48%**	jumonji domain containing 2B
C10orf116	24%	**52%**	chromosome 10 open reading frame 116
ANXA9	19%	**43%**	annexin A9
AR	19%	**33%**	androgen receptor (dihydrotestosterone receptor; Kennedy disease)
CCND1	19%	**48%**	cyclin D1 (PRAD1: parathyroid adenomatosis 1)
CCNG2	19%	24%	cyclin G2
CA12	19%	**38%**	carbonic anhydrase XII
CACNA1D	19%	**33%**	calcium channel, voltage-dependent, L type, alpha 1D subunit
CACNA2D2	19%	**43%**	calcium channel, voltage-dependent, alpha 2/delta subunit 2
DNALI1	24%	**43%**	dynein, axonemal, light intermediate polypeptide 1
AGR2	19%	**33%**	anterior gradient 2 homolog (Xenepus laevis)
GFRA1	33%	**48%**	GDNF family receptor alpha 1
HPN	19%	**43%**	hepsin (transmembrane protease, serine 1)
GREB1	19%	**38%**	GREB1 protein
MAPT	19%	**38%**	microtubule-associated protein tau
MLPH	24%	**33%**	melanophilin
KRT18	24%	**33%**	keratin 18
PTPRT	24%	**48%**	protein tyrosine phosphatase, receptor type, T
STC2	24%	**33%**	stanniocalcin 2
SCUBE2	**33%**	24%	CEGP1 protein
PTGER3	**33%**	24%	prostaglandin E receptor 3 (subtype EP3)
PDCD4	**33%**	24%	programmed cell death 4 (neoplastic transformation inhibitor)
MUC1	**33%**	29%	mucin 1, transmembrane
NPY1R	**33%**	29%	neuropeptide Y receptor Y1
FLJ20366	**38%**	24%	hypothetical protein FLJ20366
TLE3	**33%**	29%	transducin-like enhancer of split 3 (E(sp1) homolog, Drosophila)
13CDNA73	24%	29%	hypothetical protein CG003
AGTR1	24%	29%	Angiotensin II receptor, type 1
ASAH1	24%	29%	N-acylsphingosine amidohydrolase (acid ceramidase) 1
BF	24%	24%	B-factor, properdin
ENPP1	24%	29%	ectonucleotide pyrophosphatase/phosphodiesterase 1
QDPR	24%	29%	quinoid dihydropteridine reductase
C9orf116	19%	29%	chromosome 9 open reading frame 116
CYFIP2	19%	29%	cytoplasmic FMR1 interacting protein 2
GRIA2	19%	29%	glutamate receptor, ionotropic, AMPA 2
GSTM3	19%	29%	Glutathione S-transferase M3 (brain)
ACOX2	19%	29%	acyl-Coenzyme A oxidase 2, branched chain
LRIG1	19%	29%	leucine-rich repeats and immunoglobulin-like domains 1
PLAT	19%	29%	plasminogen activator, tissue
MAGED2	19%	29%	Melanoma antigen family D, 2
THRAP2	19%	29%	thyroid hormone receptor associated protein 2
MSX2	24%	24%	msh homeo box homolog 2 (Drosophila)
UGCG	24%	24%	UDP-glucose ceramide glucosyltransferase
ALCAM	19%	24%	activated leukocyte cell adhesion molecule
ALDH4A1	19%	24%	aldehyde dehydrogenase 4 family, member A1
ABCA3	24%	19%	ATP-binding cassette, sub-family A (ABC1), member 3
LOC51760	19%	24%	B/K protein
PRSS23	19%	24%	protease, serine, 23
RHOH	24%	19%	ras homolog gene family, member H
TFAP2B	19%	24%	transcription factor AP-2 beta (activating enhancer binding protein 2 beta)
WFDC2	24%	19%	WAP four-disulfide core domain 2
ANGPTL1	19%	19%	angiopoietin-like 1
BCAS1	19%	19%	breast carcinoma amplified sequence 1
CYP2B6	19%	19%	cytochrome P450, subfamily IIB (phenobarbital-inducible), polypeptide 6
EML2	19%	19%	echinoderm microtubule associated protein like 2
FLNB	19%	19%	filamin B, beta (actin binding protein 278)
GPR160	19%	19%	G protein-coupled receptor 160
LU	19%	19%	Lutheran blood group (Auberger b antigen included)
MRPS30	19%	19%	mitochondrial ribosomal protein S30
PTE2B	19%	19%	peroxisomal acyl-CoA thioesterase 2B
RERG	19%	19%	RAS-like, estrogen-regulated, growth inhibitor
RNASE4	19%	19%	ribonuclease, RNase A family, 4
RNF110	19%	19%	polycomb group ring finger 2 (MEL-18)
SEMA3C	19%	19%	sema domain, immunoglobulin domain (Ig), short basic domain, (semaphorin) 3C
SULT2B1	19%	19%	sulfotransferase family, cytosolic, 2B, member 1
TPBG	19%	19%	trophoblast glycoprotein
TPD52	19%	19%	tumor protein D52
KAL1	19%	19%	Kallmann syndrome 1 sequence

After individual Oncomine meta-analysis of 21 studies both lists of coexpressing genes, for GATA3 and ERα were compared for overlap. Overlap greater than 30% frequency (7 studies) is shown in **bold**. Overlap list is arranged by percent frequency.

### Reporter gene assays

MCF-7 Cells were grown in DMEM (minus phenol-red) with 10% charcoal-stripped FBS. SKBR3 were grown in DMEM with 10% FBS. *MUC1 *(-881 to +13) was cloned as a KpnI/XhoI fragment, and *KRT18 *(-2961 to +96) was cloned as a KpnI/BglII fragment. Both were generated by high-fidelity PCR from human genomic DNA (Roche), and were ligated into pGL4.20 (Promega). pS2 reporter has previously been described [15]. Luciferase reporter gene assays were performed using standard protocols. Here 200–400 ng reporter were transfected with 200 ng pcDNA3 or pcDNA3-GATA3, and 3U/well of β-galactosidase protein (Sigma) as transfection efficiency control. Ten nM Tamoxifen (Sigma) was incubated for 14 h prior to cell assay.

## Results and Discussion

**Figure 1:**
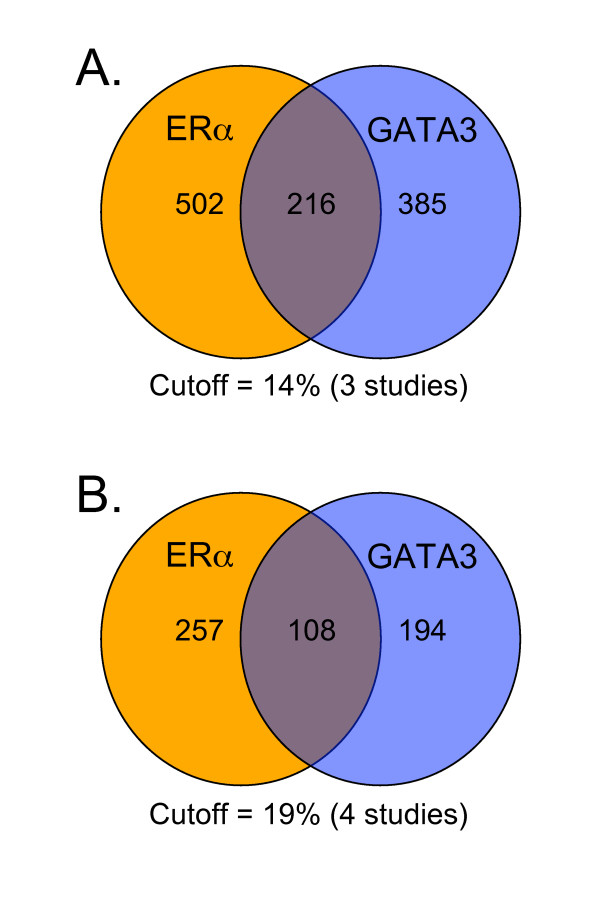
**Venn diagram showing overlap between ERα and GATA3 meta-analyses**. (A) Overlap when the frequency cutoff is 3 studies (14%). (B) Overlap when the frequency cutoff is 4 studies (19%).

Using the Oncomine™ integrated cancer profiling database GATA3 and ERα were searched for coexpressing genes [13]. Coexpression data from 21 multi-array studies was extracted and analysed, separately for ERα and GATA3. While these studies varied in cancer-types, the overwhelming majority extracted for analysis were breast-cancer based [Additional file 1 and 2]. The frequency of coexpressing genes over the 21 studies was determined and the cutoff set to 3 studies or more (3 studies = 14% frequency overlap – [see Additional file 1 and 2]). Next, to ascertain the extent GATA3 may play a role in ERα pathways the frequency coexpression lists were compared for overlap. Interestingly, there was an extensive overlap between both GATA3 and ERα lists at the cutoff of 3 studies (Figure 1A). Increasing the cutoff to 4 or more studies (almost one-fifth of the studies) did not change the relative overlap with respect to total gene numbers, with 43% of the number of ERα coexpressed genes, and 56% of GATA3 coexpressed genes represented in the overlap (Figure 1B). The overlap data with the frequency cutoff of 4 studies is shown in Table 1.

Every technique has its caveats, and the limitation here is that we are assessing the common genes that are consistently coexpressed with ERα and GATA3 over many different human cancer studies. This implies that coexpressed genes are in the same pathways as GATA3 and ERα. However, the meta-analyses can only infer association within the same pathways, and pathway coexpression at the RNA level might not necessarily translate into protein level. Nevertheless, our data are strongly supported by previous knowledge of the ERα pathway.

A recent study identified 51 genes significantly upregulated in ERα positive breast tumours, using a real-time PCR based approach [16]. Attesting to the stringency of the meta-analysis approach used here 32 of theses genes were found to overlap with the ERα coexpression list, while an identical number also overlapped with GATA3 (Table 2). This was reflected in a similar study comparing ERα over-expressed transcripts in both oligonucleotide microarray and SAGE platforms [17], where 27 genes common to the ERα pathway are represented here in our common ERα:GATA3 meta-analysis comparison [see Additional file 3]. These data not only acted as wide-ranging external validation for the individual meta-analyses, but also confirmed the extent of the involvement of GATA3 in ERα pathways.

**Table 2:** Comparison of GATA3 and ERα meta-analyses, and RT-PCR study

	**GATA3 Oncomine**	**ERα Oncomine**
ESR1	✔	✔
GATA3	✔	✔
TFF1	✔	✔
TFF3	✔	✔
FOXA1	✔	✔
XBP1	✔	✔
IL6ST	✔	✔
KRT18	✔	✔
AR	✔	✔
BCL2	✔	✔
CCND1	✔	✔
RERG	✔	✔
ERBB4	✔	✔
NAT1	✔	✔
SLC39A6	✔	✔
DNAJC12	✔	✔
HPN	✔	✔
CYP2B6	✔	✔
CA12	✔	✔
STC2	✔	✔
ACADSB	✔	✔
LRBA	✔	✔
PTPRT	✔	✔
SULT2B1	✔	✔
MYB	✔	✔
SEMA3B	✔	✔
RET	✔	✔
SLC7A2	✔	✔
RABEP1	✔	
IGFBP4	✔	
CGA	✔	
GJA1	✔	
PGR		✔
RARRES		✔
BBC3		✔
LOC255743		✔

51 genes were identified as being upregulated in ERα-positive breast tumours in a recent study by Tozlu *et al*, and are compared with the Oncomine meta-analysis lists for ERα and GATA3, showing a significant overlap. ✔ shows that this gene is represented.

Furthermore, when compared to a list of genome-wide promoters shown to be bound by ERα in MCF-7 cells [18] or on chromosomes 21 and 22 [19], 23 were identified in the ERα meta-analysis list, while 27 were identified within the GATA3 list (Table 3). This again supports both the validity of the meta-analysis technique used here, and the role of GATA3 in ERα pathways. It is also possible that the overlap would be even higher if the ERα genomic location analysis were performed on a pool of human ERα-positive breast tumour samples as opposed to a cell-culture model system. While not to detract from the power of a model system such as MCF-7 there are likely to be a great many differences between a homogeneous cell monolayer and a 3-dimensional cancer made up of a heterogeneous cell population.

**Table 3:** Comparison of GATA3 and ERα meta-analyses with previously reported binding sites (by ChIP-chip analysis)

**ERα ChIP-chip: GATA3 Oncomine**	**ERα ChIP-chip: ERα Oncomine**
**ABCA3**	**ABCA3**
ALDH3B2	**ANXA9**
**ANXA9**	BTRC
EPS8	C2
**ESR1**	CYP51A1
FLJ20152	**ESR1**
**FOXA1**	FLJ13710
**GREB1**	**FOXA1**
GTF2H2	**GREB1**
**LOC51760**	KCNAB2
**MGC11242**	**LOC51760**
MGP	MB
NAV3	**MGC11242**
NQO1	MSP
PDZK1	**SEMA3B**
PHF15	**SLC27A2**
RTN1	**SLC7A2**
**SEMA3B**	STARD10
**SLC27A2**	**STK39**
**SLC7A2**	**TFF1**
SLC7A8	**TFF3**
**STARD10**	**NRIP1**
**STK39**	RUNX1
**TFF1**	
**TFF3**	
TOMM40	
**NRIP1**	

Oncomine meta-analysis data for GATA3 or ERα was compared both to a promoter list published by Laganiere *et al*, (P = 0.05), and to a chromosome array list of 30 genes identified by Carroll *et al*. The overlap is shown and common overlap between ERα and GATA3 is shown in **bold**.

Of the 10 classifier genes previously identified in a meta-analysis of ERα, the same 4 were identified in both meta-analyses of this study (*ESR1*, *GATA3*, *FOXA1*, *SLC39A6*) [9]. Once again this adds credence to the high-quality data obtained in our current meta-analyses.

Implicating GATA3 in control of some of these gene products is a microarray experiment performed after overexpression of GATA3 in 293T cells [20]. After expression of GATA3 elevated levels of *TFF1*, *TFF3*, *KRT18*, *FOXA1*, *SLC9A3R1*, *TPD52*, *BCAS1 *were observed, all of which we identified here for both GATA3 and ERα meta-analyses. While 293T are not breast cancer cells, it raises the question of how many more of our predicted pathway partners of GATA3 would be identified if the microarray were repeated in cells such as MCF-7 which also retain high ERα expression. In the example of *SLC9A3R1 *(NHERF1) which is a putative tumour suppressor, it was shown to increase growth of 2 breast cancer cell lines when knocked down by shRNA [21]. If GATA3 does help to control expression of NHERF1 this might be one mechanism consistent with its role in the less-aggressive differentiated luminal A breast cancers. Another example is *BCAS1 *(NABC1) which is overexpressed in breast carcinomas but downregulated in colorectal tumours [22,23]. Indeed, overexpression of NABC1 did not result in changes in cell-cycle or anchorage-dependent growth properties in NIH3T3 cells, implying it may not be intrinsically oncogenic [24].

**Figure 2:**
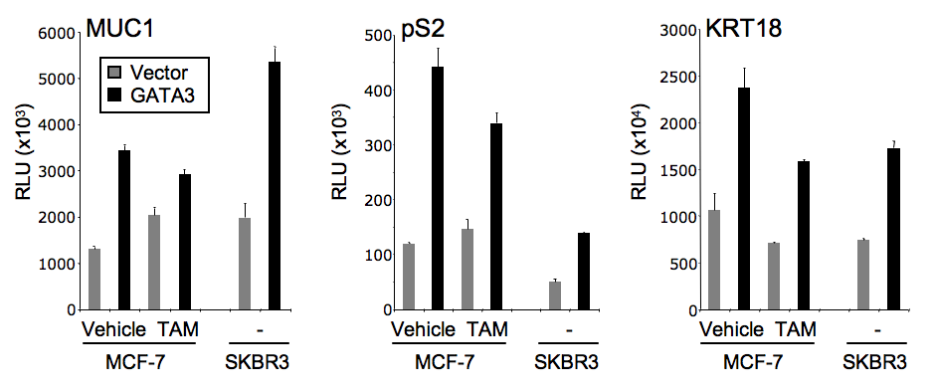
**GATA3 can activate ERα pathway promoter reporters**. GATA3 can activate MUC1, pS2, or keratin 18 promoter reporters, in ERα-positive MCF-7 cells (even in the presence of tamoxifen – TAM), or in ERα-negative SKBR3 cells.

As GATA3 is expressed in, and regulates, luminal epithelial cells and has also been shown to regulate the *MUC1 *gene it is no surprise that *MUC1 *is also mostly expressed in luminal breast epithelial cells as well as other glandular epithelia [25]. *MUC1*, when abnormally expressed, leads to a loss of both cell-extracellular and cell-cell contacts. It has also been shown that *MUC1 *levels can be regulated by estrogen and ERα can bind putative binding sites derived from the *MUC1 *promoter *in-vitro *[26]. Here we reveal that both GATA3 and ERα coexpress with *MUC1 *acting as further validation of the meta-analysis technique used here. Furthermore, transfected GATA3 can activate a *MUC1 *promoter reporter in MCF-7 cells, even in the presence of Tamoxifen i.e. independently to ERα activation. This activation could be repeated in the ERα-negative breast cancer cell line SKBR3 (Figure 2). The activation of ERα pathway genes was also observed with pS2 (*TFF1*) and *KRT18 *reporters (Figure 2). These data indicate that GATA3 can have its own impact on the ERα pathway and is not just acting indirectly via ERα.

It has also been postulated that, as the deletion of GATA3 in mammary primordia (by K14-Cre) resulted in an inability to form mammary placodes is similar to that of loss of LEF1, Msx1 and Msx2 these may all be intertwined in a transcriptional network [4,27]. It is of interest that in our present study we observe *MSX2 *coexpression both with GATA3 and ERα, which helps to support this notion.

Using the meta-analysis data presented it is easy to build up transcriptional networks such as this and all of the data presented strongly supports (1) the quality of the meta-analysis results, (2) the concept that GATA3 is firmly entrenched within ERα pathways. Future in-depth analysis of the data presented may lead to novel aspects of ERα or GATA3 regulated pathways, and help to understand the etiology of ERα-positive breast cancers, and management of their outcomes.

## Conclusion

While GATA3 has been identified previously in a meta-analysis of ERα only 10 genes were identified in total [9]. Here we give an extensive list of coexpressed ERα genes and for the first time a *reciprocal *meta-analysis for GATA3 has been performed, and the results compared for overlap. This overlap was considerable, confirming the important role of GATA3 in the ERα pathway. The vital question raised is whether GATA3 is crucial to the ERα pathway only by regulation of ERα levels, or through further control of ERα-regulated genes in concert with ERα itself. The GATA3 overexpression microarray experiment in 293T cells, and our reporter gene assays certainly implies the latter [20]. Genome-wide location analysis (ChIP-chip) of GATA3 in a well-established ERα system such as MCF-7 cells, as well as specific analysis of the ERα pathway in GATA3 conditional knockout mice will yield vital information regarding the extent that GATA3 is integral to the ERα pathway.

## Authors' contributions

BW conceived and designed the study, performed the meta-analyses, the reporter assays, and wrote the manuscript. VG critically reviewed the manuscript, and approved the final version.

## Supplementary Material

Additional file 1 GATA3 Oncomine meta-analysis. Meta-analysis results from 21 Oncomine studies shown. Coexpressing genes with GATA3 are shown with a cutoff of 3 studies (14% of the 21 studies). Click here for file

Additional file 2 ERα Oncomine meta-analysis. Meta-analysis results from 21 Oncomine studies shown. Coexpressing genes with ERα are shown with a cutoff of 3 studies (14% of the 21 studies). Click here for file

Additional file 3 External data comparison. Comparison of data to that of Abba *et al*, 2005. ERα pathway genes common to oligo microarrays, SAGE and our meta-analysis overlap. Click here for file
